# Chlorogenic acid modulates the ubiquitin–proteasome system in stroke animal model

**DOI:** 10.1186/s42826-022-00151-2

**Published:** 2022-12-21

**Authors:** Murad-Ali Shah, Ju-Bin Kang, Phil-Ok Koh

**Affiliations:** Department of Anatomy, College of Veterinary Medicine, Research Institute of Life Science, Gyeongsang National University, 501 Jinju-Daero, Jinju, 52828 South Korea

**Keywords:** Cerebral ischemia, Chlorogenic acid, Neuroprotection, Ubiquitin–proteasome system

## Abstract

**Background:**

Chlorogenic acid, a phenolic compound, has potent antioxidant and neuroprotective properties. The ubiquitin–proteasome system is an important regulators of neurodevelopment and modulators of neuronal function. This system is associated with neurodevelopment and neurotransmission through degradation and removal of damaged proteins. Activation of the ubiquitin–proteasome system is a critical factor in preventing cell death. We have previously reported a decrease in the activity of the ubiquitin–proteasome system during cerebral ischemia. This study investigated whether chlorogenic acid regulates the ubiquitin–proteasome system in an animal stroke model. In adult rats, middle cerebral artery occlusion (MCAO) surgery was performed to induce focal cerebral ischemia. Chlorogenic acid (30 mg/kg) or normal saline was injected into the abdominal cavity 2 h after MCAO surgery, and cerebral cortex tissues were collected 24 h after MCAO damage.

**Results:**

Chlorogenic acid attenuated neurobehavioral disorders and histopathological changes caused by MCAO damage. We identified the decreases in ubiquitin C-terminal hydrolase L1, ubiquitin thioesterase OTUB1, proteasome subunit α type 1, proteasome subunit α type 3, and proteasome subunit β type 4 expression using a proteomics approach in MCAO animals. The decrease in these proteins was alleviated by chlorogenic acid. In addition, the results of reverse transcription-polymerase chain reaction confirmed these changes. The identified proteins were markedly reduced in MCAO damage, while chlorogenic acid prevented these reductions induced by MCAO. The decrease of ubiquitin–proteasome system proteins in ischemic damage was associated with neuronal apoptosis.

**Conclusions:**

Our results showed that chlorogenic acid regulates ubiquitin–proteasome system proteins and protects cortical neurons from neuronal damage. These results provide evidence that chlorogenic acid has neuroprotective effects and maintains the ubiquitin–proteasome system in ischemic brain injury.

## Background

Chlorogenic acid (CGA) is a phenolic compound abundant in coffee and tea, and is well known as an antioxidant [1]. It has anti-inflammatory, antibacterial, and anti-cancer properties and also exerts neuroprotective effect [2–5]. CGA also improves sensory motor impairment in ischemic brain damage, reduces edema, improves memory loss due to cerebral ischemia, and prevents apoptosis [6, 7]. It alleviates the production of reactive oxygen species and the increase of apoptosis-related proteins in the cerebral ischemia–reperfusion animal model [8, 9]. It improves cognitive function, and reduces the risk of neurodegenerative diseases such as Alzheimer's disease [5, 10].

The ubiquitin–proteasome system is a major candidate for maintaining cell homeostasis by controlling various major processes such as cell division, apoptosis, cell signalling, and membrane penetration [11]. The system is essential for removing misfolded, mutant, and damaged proteins [12]. Protein aggregates in neurons are considered to cause neurological disorders and neuronal degeneration. The accumulation of protein aggregates due to cerebral ischemia causes neuronal cell death [13]. Accurately, cerebral ischemia induces the formation of ubiquitin-containing clusters of misfolded or damaged proteins [14]. After transient brain ischemia, the translational complex component is coagulated into an abnormal protein aggregate. This translation inhibitions lead to neuronal cell death due to irreversible aggregation of translation complex components, chaperones and protein folding enzymes [15, 16]. In addition, unbalanced ubiquitination and deubiquitination systems and damaged proteasomes are the causes of many neurodegenerative diseases [17]. The deficits of ubiquitin–proteasome system lead to protein aggregation, which causes neurodegenerative diseases. This highlights the importance of regulation of the ubiquitin–proteasome system in cerebral ischemia. Anti-oxidative and neuroprotective effects of CGA in ischemic animal models are well demonstrated [8, 9]. We previously identified changes in various ubiquitin–proteasome system proteins in ischemic brain injury caused by middle cerebral artery occlusion (MCAO) [18]. We also identified neuroprotective effects of CGA in brain damage [19]. Thus, we hypothesized that CGA regulates the ubiquitin–proteasome system in ischemic brain injury.

## Results

### Neuroprotective effects of CGA in a right MCAO animal model

**Fig. 1:**
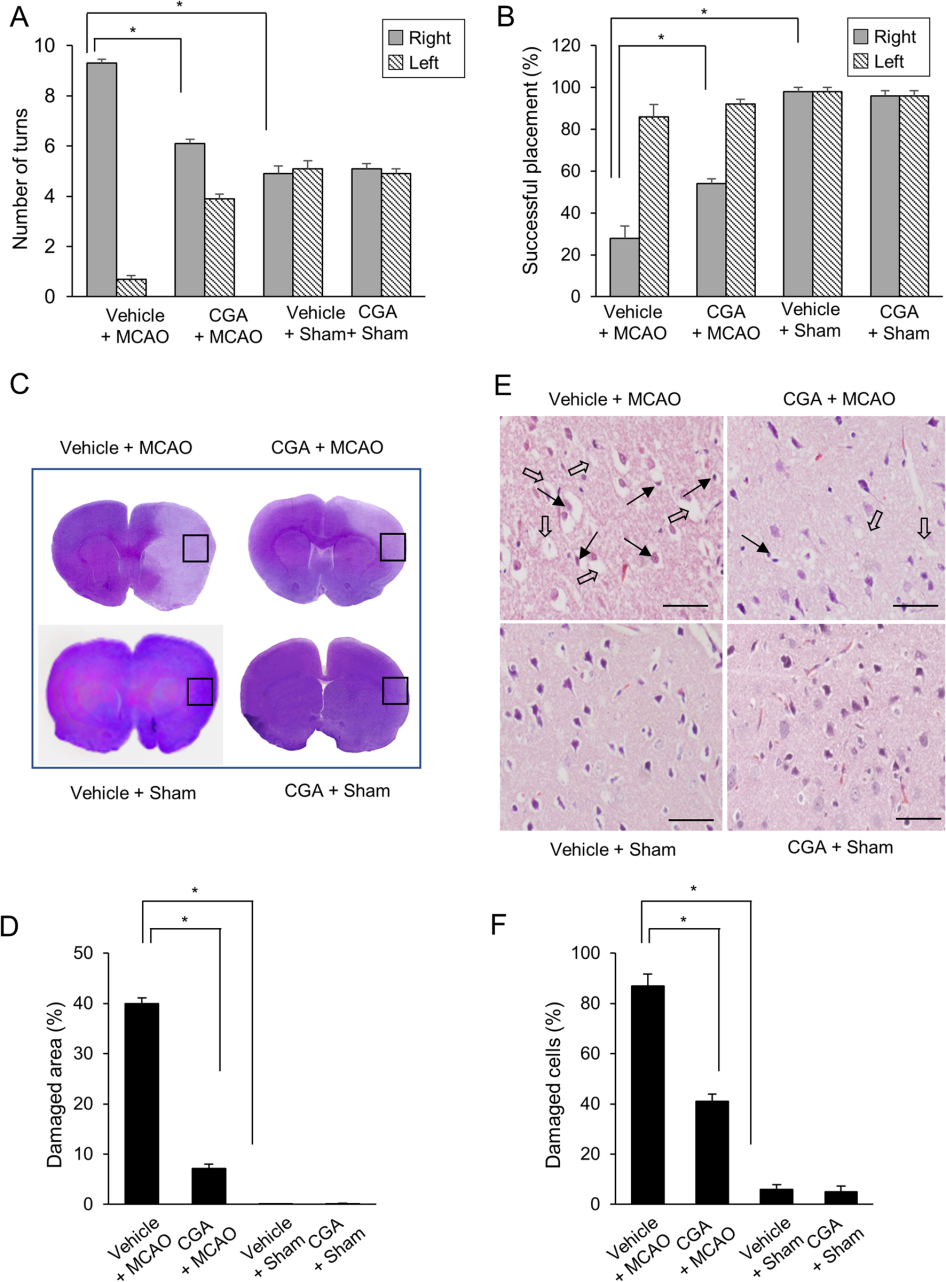
Neuroprotective effects of chlorogenic acid (CGA) in a middle cerebral artery occlusion (MCAO) animal model. Neurobehavioral corner test (**A**), vibrissae-evoked forelimb placement test (**B**), hematoxylin and eosin staining (**C**–**E**) in the vehicle + MCAO, CGA + MCAO, vehicle + sham, and CGA + sham animals. CGA attenuated MCAO-induced neurobehavioral deficits and histopathological changes. Square indicates the representative area of high magnification image. Damaged area was calculated as the ratio of infarct area to total brain area (**D**). Black arrows indicate condensed and shrunken nuclei, and white arrows indicate vacuolated and swollen forms (**E**). Scale bar = 100 μm. Data (**A** and **B**, *n* = 14; **D** and **F** *n* = 4) are represented as the mean ± S.E.M. * *p* < 0.05

Neurobehavioral deficits from right cerebral ischemia were evaluated by corner test and vibrissae-evoked forelimb placement test (Fig. 1A and B). The corner test showed a relatively high number of right turns in the vehicle + MCAO animals. Right MCAO animals are less sensitive to the stimulation of the right due to right cerebral cortical damage, so the damage to the right cerebral cortex reduces the response to the stimulation of the right vibrissae and turns the animal to the right. CGA treatment alleviated this increase. The number of right turns was 9.3 ± 0.15 and 6.1 ± 0.18 in vehicle + MCAO and CGA + MCAO animals, respectively. The number of left turns was 0.7 ± 0.15 in vehicle + MCAO and 3.9 ± 0.18 in CGA + MCAO animals (Fig. 1A). Sham animals responded equally to the stimuli of the vibrissae on both the right and left, and turn equally to the right and left. These responses were observed in sham animals regardless of vehicle or CGA treatment. The results of the vibrissae-evoked forelimb placement test showed decreased sensory motor response to right stimuli in vehicle + MCAO animals. Because MCAO animals has weak stimulus on the right vibrissae due to right cerebral cortical damage, the successful placement of right forelimb on the edge of the table is lower than opposite side. Thus, the successful placement of the right forelimbs decreased in the vehicle + MCAO animals due to the insensitivity of the right vibrissae. However, these deficits were reduced by CGA treatment, with results of 28 ± 5.10% and 54 ± 3.74% in vehicle + MCAO and CGA + MCAO animals, respectively (Fig. 1B). No deficits were found in sham animals, regardless of vehicle or CGA treatment. Hematoxylin and eosin staining revealed histopathological changes in the ischemic cerebral cortex caused by MCAO (Fig. 1C and E). The entire brain image of vehicle + MCAO animal showed the infarct area which less stained than intact area. This change was attenuated by CGA. The percentage of damaged area was 39.93 ± 1.13% and 7.14 ± 0.83% in vehicle + MCAO and chlorogenic acid + MCAO animals, respectively (Fig. 1D). Moreover, MCAO animals with vehicle treatment harbored severely damaged neurons with condensed nuclei, cytoplasmic contraction, absence of dendrites, and vacuolization. The structural damage by MCAO was reduced by CGA treatment. Normal neurons with pyramid-shaped cell bodies and large and round nuclei were observed in sham animals, regardless of vehicle or CGA treatment. The percentage of damaged cells was 87 ± 4.67% and 41 ± 2.91% in vehicle + MCAO and CGA + MCAO animals, respectively (Fig. 1F).

### Identification of changes in ubiquitin–proteasome system proteins by CGA in an MCAO animal model

**Fig. 2:**
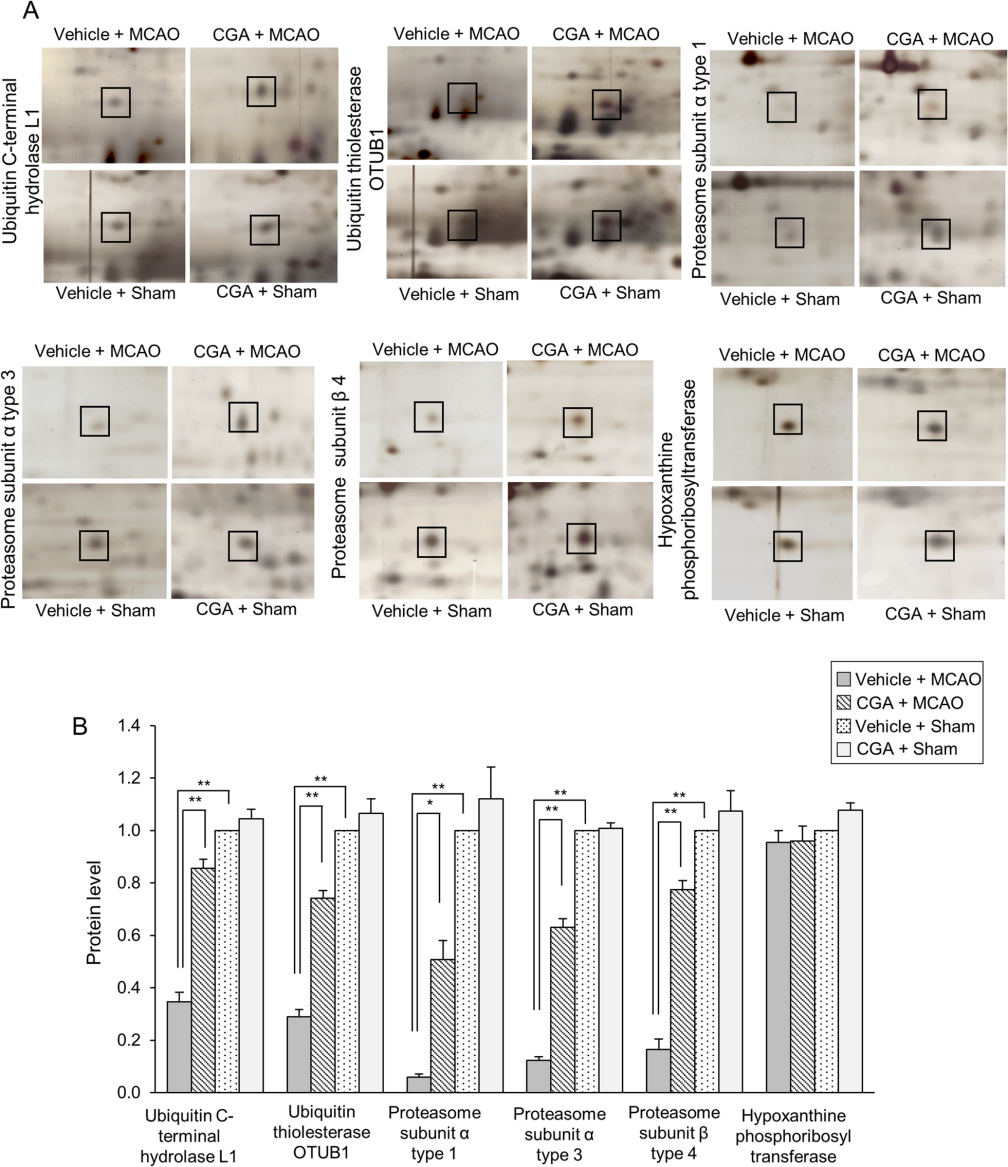
Chlorogenic acid (CGA) inhibits the decrease in ubiquitin–proteasome system proteins in the right ischemic cortex. Images of protein spots **A** of ubiquitin C-terminal hydrolase L1, ubiquitin thioesterase OTUB1, proteasome subunit α type 1, proteasome subunit α type 3, proteasome subunit β type 4, and hypoxanthine phosphoribosyltransferase in the cerebral cortex from vehicle + middle cerebral artery occlusion (MCAO), CGA + MCAO, vehicle + sham, and CGA + sham animals. MCAO significantly reduced the expression levels of the ubiquitin–proteasome system proteins and CGA treatment maintained the levels of these proteins. Each square indicates protein spots. Intensities of protein spots are represented as a ratio of intensity of each group to that of vehicle + sham group **B**. Data (*n* = 5 per group) are represented as the mean ± S.E.M. *p < 0.05, **p < 0.01

We identified changes in various proteins between vehicle- and chlorogenic-treated animals with MCAO damage. These proteins were identified by MALDI-TOF analysis. Among these proteins, we focused on ubiquitin C-terminal hydrolase L1, ubiquitin thioesterase OTUB1, proteasome subunit α type 1, proteasome subunit α type 3, and proteasome subunit β type 4. Figure 2 shows representative images of these proteins. The expression level of these proteins was measured by the intensity of the protein at those locations. The expression level of each group was evaluated by comparison to that in the vehicle + sham group. These proteins were significantly decreased in MCAO animals with vehicle treatment compared to the expression levels in sham animals. However, CGA prevented the reduction of these proteins induced by MCAO. We also identified a hypoxanthine phosphoribosyltransferase protein that did not change significantly in all experimental groups. Therefore, we considered our proteomics research to have been carried out perfectly. The expression level of each group was evaluated by the ratio of vehicle + sham group. The expression of these proteins in the vehicle + sham group and CGA + sham group was not significantly different. Ubiquitin C-terminal hydrolase L1 expression level was 0.35 ± 0.04 in vehicle + MCAO and 0.86 ± 0.03 in CGA + MCAO animals. Expression level of ubiquitin thioesterase OTUB1 was 0.29 ± 0.03 and 0.74 ± 0.03 in vehicle + MCAO and CGA + MCAO animals, respectively. Proteasome subunit α type 1 level was 0.06 ± 0.01 and 0.51 ± 0.07 in vehicle + MCAO and CGA + MCAO animals, respectively. Expression level of proteasome subunit α type 3 was 0.12 ± 0.01 and 0.63 ± 0.03 in vehicle + MCAO and CGA + MCAO animals, respectively. Proteasome subunit β type 4 level was 0.17 ± 0.04 in vehicle + MCAO and 0.77 ± 0.03 in CGA + MCAO animals. These results showed the regulation of the ubiquitin–proteasome system by CGA in cerebral ischemia.

### Reverse transcription‑PCR analysis of ubiquitin–proteasome system proteins by CGA in an MCAO animal model

**Fig. 3:**
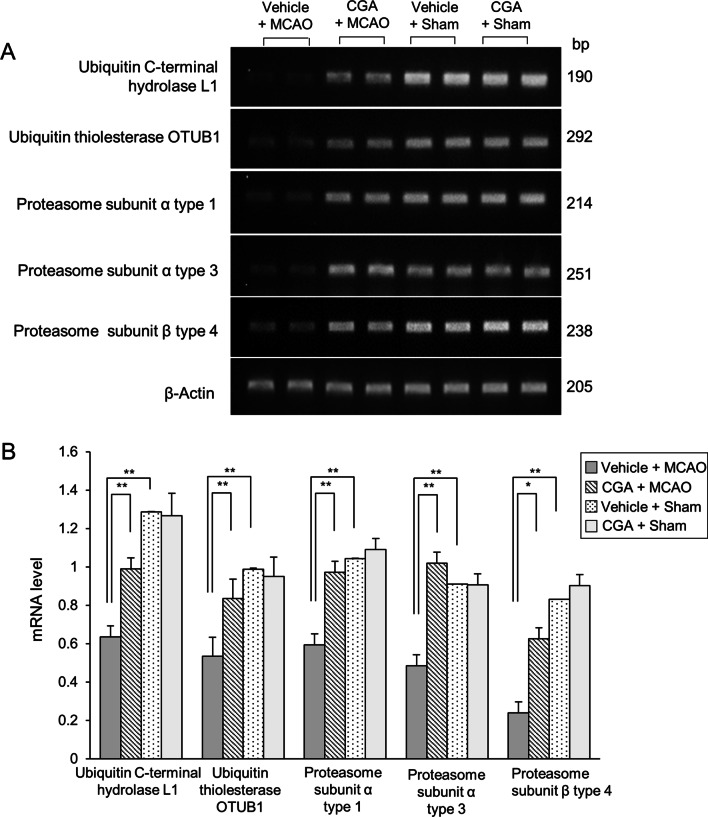
Chlorogenic acid (CGA) maintains the mRNA levels of ubiquitin–proteasome system proteins in the right ischemic cortex. Image of reverse transcription-PCR products **A** of ubiquitin C-terminal hydrolase L1, ubiquitin thioesterase OTUB1, proteasome subunit α type 1, proteasome subunit α type 3, and proteasome subunit β type 4 in the cerebral cortex from vehicle + middle cerebral artery occlusion (MCAO), CGA + MCAO, vehicle + sham, and CGA + sham animals. MCAO induced cerebral ischemia markedly decreased the mRNA levels of the ubiquitin–proteasome system proteins while treatment with CGA inhibited the MCAO-induced decrease in the mRNA levels of these proteins. Intensities of PCR product are represented as a ratio of β-actin product intensity (**B**). Data (*n* = 5 per group) are represented as the mean ± S.E.M. *p < 0.05, **p < 0.01

We used the reverse transcription-polymerase chain reaction (PCR) technique to confirm the changes in these ubiquitin–proteasome system proteins in the cerebral cortex of MCAO animals (Fig. 3). The expression of these genes was significantly decreased in MCAO-damaged animals compared to sham animals. However, CGA treatment attenuated this decline. Moreover, there was no significant difference in expression of these genes between vehicle + sham and CGA + sham animals. The expression levels of these genes were measured using the intensity of the PCR products. The mRNA level of ubiquitin C-terminal hydrolase L1 was 0.64 ± 0.03 and 0.99 ± 0.04 in vehicle + MCAO and CGA + MCAO animals, respectively. Ubiquitin thioesterase OTUB1 mRNA level was 0.53 ± 0.06 in vehicle + MCAO and 0.84 ± 0.05 in CGA + MCAO animals. The mRNA level for proteasome subunit α type 1 was 0.59 ± 0.04 and 0.97 ± 0.05 in vehicle + MCAO and CGA + MCAO animals, respectively. Proteasome subunit α type 3 mRNA level was 0.49 ± 0.03 in vehicle + MCAO and 1.02 ± 0.04 in CGA + MCAO animals. Proteasome subunit β type 4 mRNA level was 0.24 ± 0.03 and 0.62 ± 0.04 in vehicle + MCAO and CGA + MCAO animals, respectively.

## Discussion

Prior to this experiment, we confirmed the neuroprotective effect of CGA in the ischemic stroke animal model through neurobehavioral defect tests and histological staining. MCAO damage caused severe neurological disorders and histopathological changes. CGA therapy alleviated the neurobehavioral functional deficits caused by MCAO damage and protected neurons against cerebral ischemic damage. We also identified decreases in the ubiquitin–proteasome system proteins ubiquitin C-terminal hydrolase L1, ubiquitin thioesterase OTUB1, proteasome subunit α type 1, proteasome subunit α type 3, and proteasome subunit β type 4 in MCAO-induced brain damage. The decrease in these proteins was attenuated by CGA treatment in MCAO animals. Hypoxanthine phosphoribosyltransferase is known as a housekeeping gene in focal cerebral ischemic model [20, 21]. We also identified a hypoxanthine phosphoribosyltransferase protein that did not change significantly in all experimental groups. Therefore, we considered our proteomics research to have been carried out perfectly.

The ubiquitin–proteasome system is essential for regulating deubiquitinase and proteasomes. The system is associated with a variety of cell processes, including cell division, cell death, and cell signaling [11]. The system plays a role in removing proteins that are misfolded, damaged, or aggregated [12]. Ubiquitin C-terminal hydrolase L1 is a deubiquitinating enzyme that is expressed extensively in neurons and represents 1–2% of total soluble proteins in the brain [22]. It plays an important role in the cell cycle and protein degradation through cleavage of ubiquitinated peptides [23, 24]. Inhibition of ubiquitin C-terminal hydrolase L1 exacerbates impairment of synaptic transmission and synaptic plasticity that is involved in learning and memory and also increases hypoxic neuronal cell death after ischemia [25, 26]. Furthermore, the level of ubiquitin C-terminal hydrolytic enzyme L1 is reduced in Alzheimer's disease [27] and in the cerebral cortex of ischemic stroke animals [28]. We found that CGA alleviates the reduction of ubiquitin C-terminal hydrolase L1 expression due to MCAO damage. Ubiquitin C-terminal hydrolytic enzyme L1 exhibits neuroprotective effects against axon damage and oxidative stress and performs neuroprotective functions [29, 30]. Therefore, the results of this study demonstrate that the regulation of ubiquitin C-terminal hydrolase L1 expression by CGA is associated with the neuroprotective mechanism of CGA in ischemic brain injury.

Ubiquitin thioesterase OTUB1 is a deubiquitinase found in various human tissues and expressed highly in the brain [31]. Deubiquitination plays an important role in cell cycle regulation, proteasomal- and lysosomal-dependent protein degradation, and DNA repair [32–34]. However, abnormalities in deubiquitinase can lead to serious diseases such as cancer and neurological disorders [35, 36]. Misfolded or damaged proteins aggregate with ubiquitin in cerebral ischemic damage and induce neuronal cell death [13, 37]. However, inhibition of ubiquitination and deubiquitination systems can affect the degradation process, which is the cause of many neurodegenerative diseases [15–17]. The reduction in ubiquitin thioesterase OTUB1 in the cerebral cortex with MCAO damage was confirmed. We also showed that CGA alleviates the reduction of ubiquitin thioesterase OTUB1 due to MCAO damage. These results were confirmed by proteomic study and PCR analysis. The reduction of ubiquitin thioesterase OTUB1 causes incomplete deubiquitination. Our results clearly showed that CGA regulates the expression of ubiquitin thioesterase OTUB1 in ischemic brain damage, playing an important role in neuroprotection. Thus, we provide evidence that CGA regulates the expression of ubiquitin thioesterase OTUB1, and that maintaining the expression of this protein is associated with the neuroprotective function of CGA.

Proteasomes are multi-catalytic proteases found in nuclei and cytoplasm [38, 39]. The 26S proteasome is composed of a 20S catalytic core particle and 19S regulatory particles [40]. The core of the proteasome is referred to as the 20S proteasome, which consists of α- and β-subunits. These subunits regulate proteasomal protein degradation and stabilize the 20S proteasome complex [41]. Proteasomes are responsible for the degradation of proteins and have crucial roles in major cellular processes that include cell proliferation and apoptosis [39, 42]. Proteasomes decompose misfolded or damaged proteins through the protein degradation mechanism. The proteasome maintains neuronal homeostasis and mediates increases in oxidative stress and neurodegeneration [43, 44]. Because proteasomes are associated with protein degradation in most proteins, the decrease in proteasome expression causes cell destruction and denaturation, which eventually cause neurodegenerative and neurological dysfunction [41, 45]. In the current study, we showed decreases in expression of proteins proteasome subunit α type 1, proteasome subunit α type 3, and proteasome subunit β type 4 in cerebral ischemic damage. The decrease in these proteins permits increases in misfolded and ubiquitinated proteins, which cause neurological changes and disorders. We further described the alleviation of these protein reductions by CGA during cerebral ischemia. Maintenance of proteasome protein expression is important for cell survival and protection against ischemic damage. The suppression of proteasome systems in cerebral ischemia induces neuronal apoptosis and cell damage [46–48]. These data suggest that proteasome proteins are associated with the neuroprotective mechanisms of CGA against brain ischemia. CGA prevents ubiquitin-mediated degradation by controlling IκB protein in inflammatory processes [49]. It also modulates antioxidative activity through proteasome regulation, protects against degenerative neuronal damage, and regulates a neuroprotective pathway in neurodegenerative disorders [50]. CGA has a neuroprotective effect in cerebral ischemia and maintains the expression of proteasome proteins and deubiquitinating enzymes. We demonstrate that activation of the proteasome and deubiquitinating enzymes is associated with the protective mechanism of CGA against ischemic brain damage. However, more research is needed to fully explore the neuroprotective effects of CGA in ischemic stroke animal models.

## Conclusions

Our results showed that MCAO damage causes neurobehavioral disorders, and that CGA attenuates neurodegenerative disorders. CGA alleviates the reduction of ubiquitin C-terminal hydrolase L1, ubiquitin thioesterase OTUB1, proteasome subunit α type 1, proteasome subunit α type 3, and proteasome subunit β type 4 in ischemic brain injury, all of which are part of the ubiquitin–proteasome system. Thus, our findings demonstrate that CGA exerts neuroprotective effects by controlling the ubiquitin–proteasome system in MCAO damage.

## Methods

### Animal grouping and drug injection

Adult Sprague Dawley rats (male; 200–220 g) were purchased from Samtako Bio (Osan, South Korea). Animals were kept in laboratory animal rooms for a week before grouping and drug injection to adapt to the new environment (12 h bright/12 h dark cycle). All the experimental procedures were conducted in accordance with the rules of the Institutional Animal Care and Use Committee of Gyeongsang National University (GNU-220222-R0021). The animals were randomly divided into four groups: vehicle + sham, CGA + sham, vehicle + MCAO, and CGA + MCAO. The animals were injected in the abdominal cavity with vehicle or CGA 2 h before MCAO. Phosphate buffered saline (PBS) was used as a solvent for CGA, and the vehicle group was injected only with PBS without drugs. CGA (Sigma-Aldrich, St. Louis, USA) was administered at 30 mg/kg according to previous criteria [6]. Food and water were supplied freely to all animals during the experiment. We assigned 14 rats per group for the following experiments: hematoxlyin and eosin staining (*n* = 4 for each group), two-dimensional gel electrophoresis (*n* = 5 for each group), and reverse transcription PCR (*n* = 5 for each group), respectively. Neurobehavioral tests were conducted in all animals.

### Middle cerebral artery occlusion

MCAO was performed with a previously reported procedure [51]. Zoletil (50 mg/kg, Virbac, Carros, France) was injected intramuscularly for induction of anesthesia in all animals. The animals were carefully operated on the heating pads to prevent hypothermia in a supine position. A midline incision of the neck was performed and the right common carotid artery (CCA), external carotid artery (ECA), and internal carotid artery (ICA) were properly exposed and separated from nearby tissues and nerves. The right CCA was temporarily blocked by microvascular clamp, and the proximal end of the ECA was ligated. A cut was given to the ECA and a 4–0 nylon suture rounded by heat was inserted into the severed ECA until resistance was felt. The length of the inserted nylon suture was 22–24 mm. The blunt end of nylon blocked the origin of middle cerebral artery and blocked blood flow to the brain. The nylon suture was ligated with the proximal end of the ECA with a silk suture. The incisied region of neck was closed with a black silk suture and the animals were kept back in cages. After 24 h of MCAO, rats were euthanatized with Zoletil and brains were collected for further experimental studies.

### Corner test

The corner test was performed for sensory-motor asymmetry evaluation with two whiteboards (30 × 20 × 1 cm^3^) vertically maintained at a 30º angle to each other [52]. A space was maintained at the entrance and end of the boards so that animals may move ahead to the corner without obstacles. The test was started by keeping the animals at the wide side of the boards allowing them to move to the corner. When the animals reach the corner, their vibrissae was touched the side of the board and they turn right or left side. The experiment was repeated 10 times and the number of left or right turn was recorded. Animals were trained for seven days before MCAO surgery for the corner test and animals with the same rate of right and left turns were selected for this study.

### ***Vibrissae-evoked forelimb placement test***

Vibrissae-evoked forelimb placement test was performed for the evaluation of sensory-motor function as previously reported method [53]. Animals were taken out of their cages and kept on a table before the experiment to relax their muscles and avoid any unnecessary struggling during the test. They were grabbed from the torsos and allowed to move their legs freely. The left or right vibrissae was rubbed with the edge of a table and the sensory-motor deficit was observed. When the vibrissae touched the edge of the table, animals extend and place their right or left forelimb on the edge of the table. The experiment was conducted 10 times on each animal and successful placement of the forelimb was recorded. Animals were trained for the test for five days before MCAO surgery.

### Hematoxylin and eosin staining

Whole brains from skulls were immediately removed and fixed in a 4% paraformaldehyde solution. Brains tissues were sliced into sections with a brain matrix, dehydrated with graded ethyl alcohol series (70% to 100%), and cleaned with xylene. Tissues were kept for 1 h in paraffin tank with vacuum device, embedded in paraffin, and placed on cold plate to solidify. This process was carried out using a paraffin embedding center (Leica, Wetzlar, Germany). Paraffin blocks were cut into 4 µm sections using a rotary microtome (Leica). Paraffin ribbons were mounted onto glass slides and dried on slide warmer overnight (Thermo Fischer Scientific, Waltham, MA, USA). Tissue slides were deparaffinized in xylene and rehydrated in graded ethyl alcohol series (100–70%). They were dipped in tap water to remain hydrated, treated with hematoxylin solution (Sigma-Aldrich) for 10 min, and washed with running tap water for 10 min. They were dipped in a 1% hydrochloric acid solution manufactured in 70% ethyl alcohol for tissue differentiation, and 1% ammonia solution for tissue normalization. Tissue slides were stained with eosin solution (Sigma-Aldrich) for 1 min and dehydrated with graded ethyl alcohol series (70–100%). They were cleaned with xylene and cover-slipped with permount mounting medium (Thermo Fischer Scientific). Stained tissues slides were observed using a light microscope (Olympus, Tokyo, Japan) and images were taken from the right cerebral cortex.

### Two-dimensional gel electrophoresis

Right cerebral cortex tissues were separated from the whole brain and kept in – 70 °C. We prepared proteins from each animal per group and the extracted proteins were loaded on IPG gel strips each. First, the right cerebral cortex tissue was homogenized in lysis buffer (8 M urea, 4% CHAPS, 0.2% Bio-Lyte, 40 mM Tris–Hcl). The homogenate was centrifuged at 15,000 rpm at 4 °C for 30 min and the supernatants were collected. The collected sample was precipitated in 10% trichloroacetic acid for 30 min at room temperature and centrifuged. The protein pellets were washed with 1 M Tris–Hcl (pH 7.6) and dried at room temperature. Then, lysis buffer [8 M urea, 4% CHAPS, 0.2% Bio-Lyte, 40 mM Tris–Hcl, 1% (v/v) pharmalytes, 2 µg/ml dithiothreitol (DTT)] was added to the pellets. The mixtures were sonicated for 3 min and maintained for 1 h at room temperature. They were centrifuged and the supernatant was collected from each sample. The optical density for each sample was measured with a Bradford protein analysis kits (Bio-Rad, Hercules, CA, USA) as directed by the manufacturer. A total of 50 μg of protein samples were mixed with a rehydration solution (8 M urea, 2% CHAPS, 20 mM DTT, 0.5% IPG buffer, bromophenol blue) overnight at room temperature and loaded on IPG gel strips (Immobiline DryStrip, pH 4–7, 17 cm, Bio-Rad) for overnight at room temperature for first dimensional iso-electric focusing. The isoelectric focusing was performed using an Ettan IPGphor 3(GE Healthcare, Uppasala, Sweden) with the following voltage: 250 V for 15 min, 10,000 V for 3 h, and then 10,000 V to 50,000 V. For second-dimensional electrophoresis, IPG strips were reacted with equilibration buffer [6 M urea, 30% glycerol, 2% sodium dodecyl sulfate, 50 mM Tris–Hcl, bromophenol blue] containing 1% DTT for 10 min, and then were incubated with equilibration buffer containing 2.4% iodoacetamide for 10 min. Strips were loaded into gradient gels (7.5–17.5%) and gel electrophoresis was performed with Protein-II XI electrophoreses equipment (Bio-Rad) at 10 mA until the blue line went down to the bottom of the gel. When electrophoresis was completed, gels were carefully removed from the glass plate and fixed in a fix solution (12% acetic acid in 50% methanol) for 2 h at room temperature. The gels were washed with 50% ethanol for 20 min, sensitized by reacting with 0.2% sodium thiosulfate for 10 min, and washed with distilled water. For silver staining, the gels were stained with a silver stain solution (0.2% silver nitrate, 0.75 ml/L 37% formaldehyde) for 20 min, and washed twice with distilled water. The stained gels were developed in a developing solution (2% sodium carbonate, 0.5 ml/L 37% formaldehyde) for approximately 5 min until each protein spot on the gel was visible. They were reacted with a stop solution (1% acetic acid) for 15 min to stop developing reaction and scanned with Agfar ARCUS 1200™ (Agfar-Gevaert, Mortsel, Belgium) scanner. Each gel image was analyzed using PDQuest 2-DE analysis software and proteins with differential expression among groups were selected.

### Matrix-assisted laser desorption ionization time-of-flight (MALDI-TOF) mass spectrometry

The selected protein spots were cut from the gel and treated with a destaining solution (30 mM potassium hexacyanoferrate, 100 mM sodium thiosulfate). The gel spots were washed with a washing solution (50% methanol and 10% acetic acid) to remove silver stain solution. They were dehydrated with 50 mM of ammonium bicarbonate and acetonitrile, dried in a vacuum centrifuge (Biotron, Seoul, Korea), and then incubated in a reduction solution (10 mM DTT in 0.1 M ammonium bicarbonate) at 56 °C for 45 min. They were dehydrated in 0.1 M ammonium bicarbonate and acetonitrile, and dried in the vacuum centrifuge for 20 min. The dried spots were digested in a digestion solution (12.5 ng/ml trypsin, 0.1% octyl beta-D glycopyranside in 50 mM ammonium bicarbonate) at 37 °C overnight and reacted with an extraction buffer (1% trifluoroacetic acid in 66% acetonitrile) to collect digested proteins. The extracted proteins were centrifuged in a vacuum centrifuge for 2 h. For the identification of specific proteins, two solutions were prepared by melting cellulose with an equal amount of acetone and mixing alpha-cyano-4-hyroxycinnamic acid in acetone. The resultant solutions were mixed at a ratio of 1:4 and the final matrix solution was made of a calibrants (angiotensin and neurotensin, 2 µl/ml). The extracted dried proteins were mixed with extraction buffer and matrix solution by pipetting and loaded to a MALDI-TOF plate. After drying on MALDI-TOF plate, MALDI-TOF was performed using a Voyager-DE STR (Applied Biosystem, Foster City, CA, USA). We analyzed the peak results online on the NCBI (http://www.matricscience.com/cgi/searchform.p1?FORMVER=2&SEARCH=PMF) and MS-FIT (http://prospector.ucsf.edu/prospector/cgi-bin/msform.cgi?form=msfitstandard) websites.

### Reverse transcription-polymerase chain reaction

The right cerebral cortex tissue was used and total RNA was extracted by homogenizing with Trizol Reagent (Life Technologies, Rockville, MD, USA) according to the manufacturer's instructions.Total RNA (1 μg) of each sample was used for the synthesis of single stranded complementary DNA using Superscript III first-strand system (Invitrogen, Carlsbad, CA, USA) under the manufacturer's instructions. The amplification of target genes was performed using the following primers: ubiquitin C-terminal hydrolase L1, forward primer: 5′-TGAAGCAGACCATCGGGAAC-3′, reverse primer: 5′-GAGTCATGGGCTGCCTGAAT-3′; ubiquitin thiolesterase OTUB1, forward primer: 5′-GCGACCACATCCACATCA-3′, reverse primer: 5′-ATGACCATTTACAACCACA-3′; proteasome subunit α type 1, forward primer: 5′-CCAACACAGCGATATGGCCG-3′, reverse primer: 5′-CTCTCCAGGTAAGTGCGAGC-3′; proteasome subunit α type 3, forward primer: 5′-GGCACTGGGTATGACCTGTC-3′ reverse primer: 5′-AAGGAACGAGCATCTGCCAA-3′; proteasome subunit β type 4, forward primer: reverse primer: β-actin, forward primer: 5′-TACAACCTTCTTGCAGCTCCTC-3′ reverse primer: 5′-CCTTCTGACCCATACCCACC-3′. Amplification with PCR was performed as following manual; denaturation at 94 °C for 5 min; 30 cycles of denaturation step at 94 °C for 30 s, annealing step at 54 °C for 30 s, and elongation step at 72 °C for 1 min; and a final extension at 72 °C for 10 min. The PCR product was electrophoresed in a 1% agarose gel and observed under ultraviolet light. Images were taken and the intensity of bands was measured with Image J (Media Cybernetics, Rockville, MD, USA) computer-based software.

### Statistical analysis

All data given is represented as average ± standard error of means (S.E.M.). The data from all groups were analyzed by two-way analysis of variance (ANOVA) followed by *post-hoc* Scheffe’s test. A *p* value of less than 0.05 was considered to be statistically significant.

## Data Availability

The data that support the findings of this study are available on request from the corresponding author on reasonable request.

## Abbreviations

CCA: Common carotid artery
CGA: Chlorogenic acid
DTT: Dithiothreitol
ECA: External carotid artery
ICA: Internal carotid artery
IEF: Isoelectric focusing
IPG: Immobilized pH gradient
MALDI-TOF: Matrix-assisted laser desorption ionization time-of-flight
MCAO: Middle cerebral artery occlusion
PBS: Phosphate buffered saline
PCR: Polymerase chain reaction
